# Optimized treatment strategy for early-stage medium/high-risk hormone receptor-positive/HER2-negative breast cancer

**DOI:** 10.1097/JS9.0000000000001779

**Published:** 2024-06-13

**Authors:** Zhao Bi, Xiao Sun, Jia-Xian Zhao, Peng Chen, Tian-Yi Wang, Tong-Yue Ren, Yong-Sheng Wang

**Affiliations:** a Shandong Cancer Hospital and Institute, Shandong First Medical University and Shandong Academy of Medical Sciences, Jinan, Shandong; b Department of Nuclear Medicine, The Fourth Hospital of Harbin Medical University, Harbin, Heilongjiang, People’s Republic of China

## The role of multigene signature testing in early-stage medium/high-risk hormone receptor-positive/HER2-negative (HR+/HER2−) breast cancer

Early-stage, medium/high-risk HR+/HER2− breast cancer refers to patients with stages II–III, or at least one risk factor (i.e. histological grade 3, lympho-vascular invasion, Ki-67 >20%, high gene signature risk score). For patients with HR+/HER2− breast cancer, neoadjuvant chemotherapy can only offer little benefit of downstage in tumor, especially for axillary lymph nodes. At the same time, neoadjuvant chemotherapy does not provide sufficient prognostic information. It offers limited benefits to guide postoperative adjuvant therapy for patients with HR+/HER2− breast cancer, so surgery might be recommended as the initial step^1,2^. Subsequent treatment plans are determined based on the postoperative pathological results. Current guidelines advise conducting a multigene signature test when formulating postoperative treatment plans^3–5^. Multigene signature testing tools, such as Oncotype DX (21-gene signature test), Mammprint (70-gene signature test), and PAM50, assess the recurrence risk for HR+/HER2− breast cancer patients to ascertain whether they would benefit from adjuvant chemotherapy, thereby aiding in decision-making for their treatment^6–8^. Gene signature testing is increasingly used in patients with early-stage HR+/HER2− breast cancer. National Comprehensive Cancer Network, American Society of Clinical Oncology, and Chinese Society of Clinical Oncology guidelines recommend that treatment for patients with negative axillary lymph nodes or postmenopausal patients with 1–3 positive axillary lymph nodes should be individualized based on the gene signature testing results^3–5^. However, adjuvant chemotherapy is still recommended for patients with more than four positive lymph nodes.

Patients need to be comprehensively evaluated according to clinical risk and genetic testing risk to guide postoperative adjuvant treatment. The clinical risk is principally based on the patient’s clinicopathological characteristics, such as tumor size and lymph node metastasis, etc. Risk of gene signature refers to the use of genetic testing to assess the risk of tumor recurrence. The treatment strategy might be varied when the clinical evaluation shows medium/high risk and genetic testing results indicate low risk. The current conventional treatment strategy involves avoiding chemotherapy and opting solely for endocrine therapy, which has fewer adverse effects than chemotherapy. However, genetic testing provides an overall assessment of patient’s risk of recurrence and prognosis, which is based on the average sensitivity of a cohort and not on the endocrine treatment sensitivity of an individualized population. Not all patients are sensitive to endocrine therapy. For patients with primary/secondary endocrine resistance, avoiding chemotherapy in favor of endocrine therapy might lead to insufficient treatment according to the results of genetic testing alone, ultimately resulting in breast cancer recurrence. Therefore, it is potentially problematic to base treatment decisions solely on genetic testing for early-stage medium/high-risk HR+/HER2− breast cancer.

## Neoadjuvant endocrine therapy (NET) offers a more precise treatment strategy for patients with early-stage medium/high-risk HR+/HER2− breast cancer

### The efficacy of NET can be evaluated by monitoring the Ki-67 index

Currently, the main clinical aim of NET is to evaluate the efficacy of endocrine therapy effectively. At the same time, it can also downstage HR+/HER2− breast cancers so that mastectomy might be avoided or to achieve operability in previously inoperable cancers. NET can effectively control the disease progression in patients who are sensitive to endocrine therapy. However, not all patients are sensitive to NET. In cases where patients exhibit primary endocrine therapy insensitivity, persisting with endocrine therapy might result in breast cancer recurrence. Therefore, evaluating the effectiveness of endocrine therapy becomes imperative in such scenarios. The WSG-ADAPT study enrolled patients with HR+/HER2− breast cancer with 0–3 positive lymph nodes who received preoperative endocrine therapy for 3 weeks, with subsequent treatment decisions based on the response to Ki-67 levels. The results indicate that the Ki-67 level decreased to 10% or less after receiving NET in patients with a risk score of 12–25. The 5-year disease-free survival (DFS) and overall survival (OS) for these patients were 95.6% and 97.3%, respectively. However, patients with higher Ki-67 levels after NET showed a higher risk of recurrence^9^. The POETIC trial also demonstrated that both lower initial and decreased Ki-67 levels following NET were indicative of improved clinical responses and prognoses^10^. A decrease in the Ki-67 values suggests that these patients are sensitive to endocrine therapy. The efficacy of NET can be evaluated by monitoring the Ki-67 index. In cases of NET failure/minimal effect, better disease control may be achieved by switching to chemotherapy.

### CDK4/6 inhibitor intensive treatment can significantly improve the prognosis of early-stage medium/high-risk HR+/HER2− breast cancer

Adjuvant endocrine therapy combined with intensive CDK4/6 inhibitor treatment can significantly improve the prognosis of early-stage medium/high-risk HR+/HER2− breast cancer patients. The monarchE and NATALEE studies achieved breakthrough results^11,12^. In the monarchE study, patients with medium/high-risk HR+/HER2− breast cancer saw sustained benefits in invasive DFS (iDFS) following treatment with abemaciclib in addition to endocrine therapy. The experimental group showed an iDFS of 83.6% compared to 76.0% in the control group at the 5-year mark, although the absolute benefit in iDFS was only 7.6%. The latest data for the NATALEE study^12^ showed that the 3-year absolute iDFS benefit in the experimental and control groups was only 3.3%^11,12^. Therefore, not all patients can truly benefit from CDK4/6 inhibitor intensive therapy. Considering the adverse events and economic health benefits of CDK4/6 inhibitors, it is necessary to individually screen the patients who truly benefit from intensive adjuvant therapy with CDK4/6 inhibitors to avoid undertreatment/overtreatment.

### NET could select patients who were sensitive to CDK4/6 inhibitors

NET functions as an in-vivo sensitivity assay to effectively assess responsiveness to endocrine therapy. It pinpoints individuals who are insensitive to these treatments and assists in tailored optimization of adjuvant therapy. For patients who are sensitive to endocrine therapy, there is an opportunity for exemption from adjuvant intensive therapy with CDK4/6 inhibitors. Patients with primary resistance to endocrine therapy are recommended to augment treatment with CDK4/6 inhibitors. Therefore, we conduct an ‘adaptive design’ clinical trial (clinical trial: NCT05809024) in order to identify the suitable population for the addition of intensive CDK4/6 inhibitor therapy in the adjuvant phase. Patients received 2 weeks of NET with aromatase inhibitors (AIs) followed by a repeat biopsy to assess the Ki-67 index. The patients were then grouped according to the Ki-67 level: in group A, patients underwent surgery or continued AIs until surgery if the Ki-67 was less than 10% after treatment; in group B, if the Ki-67 was still at least 10% after treatment, patients received AIs treatment combined with CDK4/6 inhibitors for one to two cycles. This was followed by per-cycle core needle biopsy to assess the Ki-67 changes, after which the patients underwent surgery or continued combination therapy until the operation. Up to now, a total of 50 patients have been enrolled in this study and the Ki-67 indexes have all decreased after the AI treatment. Among them, 40% of the patients still had a Ki-67 index greater than 10%, while the remaining 60% of patients showed a Ki-67 index 10% or less. Among patients with the Ki-67 index greater than 10%, they received CDK4/6 inhibitor treatment for one to two cycles. There, 28.6% of them still had the Ki-67 index greater than 10% after treatment. In this trial, we screened patients resistant to primary AIs by a short cycle of NET, and 71.4% of these patients had the Ki-67 index decreased after CDK4/6 inhibitor treatment. It indicated that these patients were sensitive to CDK4/6 inhibitors. Meanwhile, the other 28.6% of patients could not benefit from CDK4/6 inhibitors. Further translational studies are exploring the resistance mechanism in patients who are resistant to CDK4/6 inhibitors.

Therefore, not all patients could truly benefit from CDK4/6 inhibitors. Considering the adverse events and economic health benefits of CDK4/6 inhibitors, it is necessary to grasp the indication of CDK4/6 inhibitors intensive adjuvant therapy to avoid undertreatment/overtreatment. The ‘adaptive design’ can adjust the treatment regimen in time according to the change of Ki-67 and then select appropriate patients for (neo)adjuvant CDK4/6 inhibitors or adjuvant chemotherapy.

## Immunotherapy may become a new treatment strategy for early medium/high-risk HR+/HER2− breast cancer

Immunotherapy has changed the diagnosis and treatment pattern of solid tumors and has become one of the important treatment methods. The recent results of KEYNOTE-756^13^ and CheckMate 7FL^14^ are a breakthrough in neoadjuvant immunotherapy for HR+/HER2− patients. The KEYNOTE-756 study mainly assesses the efficacy and safety outcome between the addition of the pembrolizumab to neoadjuvant chemotherapy vs. neoadjuvant chemotherapy alone in patients with early HR+/HER2− disease. The primary study endpoint was pathological complete response (pCR). The group receiving both pembrolizumab and neoadjuvant chemotherapy presented a pCR of 24.3% (95% CI, 21.0–27.8%), compared to a pCR of 15.6% (95% CI, 12.8–18.6%) in the group undergoing neoadjuvant chemotherapy. These results suggested that patients with HR+/HER2− breast cancer may benefit from neoadjuvant immunotherapy^13^. CheckMate 7FL^14^ was also a prospective, randomized, multicenter, double-blind, placebo-controlled trial. The addition of nivolumab to neoadjuvant chemotherapy significantly improved pCR rates in the overall population. The pCR rate was also significantly improved in patients with residual cancer burden scores 0–1, and PD-L1-positive patients benefited more from neoadjuvant nivolumab^14^. The IMpassion 030 trial investigated the value of adding atezolizumab to standard anthracycline and taxane-based adjuvant chemotherapy in triple-negative breast cancer. The primary endpoint was iDFS with the intent to treat the population. In this trial, 2199 patients were enrolled and randomly assigned to either the atezolizumab+ chemotherapy arm or chemotherapy−only arm, with an iDFS event of 11.5% and 10.2% (*P*=0.37), with the OS events of 5.5% and 4.5% (*P*>0.05), respectively. Given the negative results in the adjuvant immunotherapy of the IMpassion 030 trial and positive results in the neoadjuvant immunotherapy of KEYNOTE-756 and CheckMate 7FL, the immunotherapy needs to be moved forward to the neoadjuvant phase. Therefore, neoadjuvant immunotherapy might be a game-changer for patients with HR+/HER2− breast cancer^15^. Implementing neoadjuvant immunotherapy in patients with HR+/HER2− breast cancer is a challenge for precision treatment, and the suitable population still needs to be carefully screened^16^.

In conclusion, we want to propose an ‘adaptive design’ approach for early-stage medium/high-risk HR+/HER2− patients. Patients should initially undergo NET. Subsequently, based on the evaluation of the Ki-67 index, patients who are unable to benefit from NET or are insensitive to NET can be identified. Finally, these patients could receive neoadjuvant chemotherapy combined with immunotherapy to achieve better treatment outcomes.

## Ethical approval

Not applicable.

## Source of funding

Shanghai Anti-cancer and Anti-cancer Development Foundation (CYBER-2022-002) and China Postdoctoral Science Foundation (Grant No. 2022M721987).

## Conflicts of interest disclosure

The authors declare no conflicts of interest.

## Author contribution

Z.B., T.-Y.R., and Y.-S.W. X.S., J.-X.Z., and P.C.: wrote the first draft of the manuscript; T.-Y.W.: revised the manuscript. All authors contributed to the study’s conception and design and commented on previous versions of the manuscript. In addition, all authors read and approved the final manuscript.

## Research registration unique identifying number (UIN)

Our paper is not clinical research.

## Guarantor

Zhao Bi and Yong-Sheng Wang.

## Data availability statement

The datasets generated during and/or analyzed during the current study are available from the corresponding author on reasonable request. The corresponding author had full access to all the data in the study and had final responsibility for the decision to submit for publication.

## References

[1] Spring LM Gupta A Reynolds KL. Neoadjuvant endocrine therapy for estrogen receptor-positive breast cancer: a systematic review and meta-analysis. JAMA Oncol 2016;2:1477–1486. 27367583 10.1001/jamaoncol.2016.1897 PMC5738656

[2] Harbeck N Gnant M. Breast cancer. Lancet 2017;389:1134–1150. 27865536 10.1016/S0140-6736(16)31891-8

[3] Brackstone M Baldassarre FG Perera FE. Management of the axilla in early-stage breast cancer: Ontario Health (Cancer Care Ontario) and ASCO Guideline. J Clin Oncol 2021;39:3056–3082. 34279999 10.1200/JCO.21.00934

[4] Gradishar WJ Moran MS Abraham J. Breast Cancer, Version 3.2022, NCCN Clinical Practice Guidelines in Oncology. J Natl Compr Canc Netw 2022;20:691–722. 35714673 10.6004/jnccn.2022.0030

[5] Wang YZ Yin YM Jiang ZF. Interpretation of updated key points of Chinese Society of Clinical Oncology (CSCO) Breast Cancer Guidelines 2023. Chin J Oncol Surg 2023;15:209–213.

[6] Kalinsky K Barlow WE Gralow JR. 21-Gene assay to inform chemotherapy benefit in node-positive breast cancer. N Engl J Med 2021;385:2336–2347. 34914339 10.1056/NEJMoa2108873 PMC9096864

[7] Cardoso F van’t Veer LJ Bogaerts J. 70-Gene signature as an aid to treatment decisions in early-stage breast cancer. N Engl J Med 2016;375:717–729. 27557300 10.1056/NEJMoa1602253

[8] Prat A Lluch A Turnbull AK. A PAM50-based chemoendocrine score for hormone receptor-positive breast cancer with an intermediate risk of relapse. Clin Cancer Res 2017;23:3035–3044. 27903675 10.1158/1078-0432.CCR-16-2092 PMC5449267

[9] Nitz UA Gluz O Kümmel S. Endocrine therapy response and 21-gene expression assay for therapy guidance in HR+/HER2– early breast cancer. J Clin Oncol 2022;40:2557–2567. 35404683 10.1200/JCO.21.02759

[10] Smith I Robertson J Kilburn L. Long-term outcome and prognostic value of Ki67 after perioperative endocrine therapy in postmenopausal women with hormone-sensitive early breast cancer (POETIC): an open-label, multicentre, parallel-group, randomised, phase 3 trial. Lancet Oncol 2020;21:1443–1454. 33152284 10.1016/S1470-2045(20)30458-7 PMC7606901

[11] Johnston SRD Toi M O’Shaughnessy J. Abemaciclib plus endocrine therapy for hormone receptor-positive, HER2-negative, node-positive, high-risk early breast cancer (monarchE): results from a preplanned interim analysis of a randomised, open-label, phase 3 trial. Lancet Oncol 2023;24:77–90. 36493792 10.1016/S1470-2045(22)00694-5 PMC11200328

[12] Slamon D Lipatov O Nowecki Z. Ribociclib plus endocrine therapy in early breast cancer. N Engl J Med 2024;390:1080–1091. 38507751 10.1056/NEJMoa2305488

[13] Cardoso F O’Shaughnessy J McArthur H. Phase 3 study of neoadjuvant pembrolizumab or placebo plus chemotherapy, followed by adjuvant pembrolizumab or placebo plus endocrine therapy for early-stage high-risk ER+/HER2– breast cancer: KEYNOTE-756. SABCS 2023:GS01–GS02.

[14] Loi S Curigliano G Salgado R. Biomarker results in high-risk estrogen receptor-positive, human epidermal growth factor receptor 2-negative primary breast cancer following neoadjuvant chemotherapy±nivolumab: an exploratory analysis of CheckMate 7FL. Cancer Res 2024;84:GS0101.

[15] Ignatiadis M Bailey A McArthur H. Adding atezolizumab to adjuvant chemotherapy for stage II and III triple-negative breast cancer is unlikely to improve efficacy: interim analysis of the ALEXANDRA/IMpassion030 phase 3 trial. Cancer Res 2024;84:GS01–GS03.

[16] Ren T-Y Bi Z. Exploration of immune checkpoint inhibitors in neoadjuvant therapy of early-stage breast cancer. Int J Surg 2024;110:2479–2480. 38241339 10.1097/JS9.0000000000001099 PMC11020052

